# Fracture-Dislocation Dorsal of the Proximal Interphalangeal Joint: A Case Report and Focus on Volar Plate Injuries

**DOI:** 10.7759/cureus.47663

**Published:** 2023-10-25

**Authors:** Mohammed Barrached, Saber Zari, Adnane Lachkar, Najib Abdeljaouad, Hicham Yacoubi

**Affiliations:** 1 Department of Traumatology and Orthopedic, Mohammed VI University Hospital, Faculty of Medicine and Pharmacy, Mohammed Ist University, Oujda, MAR

**Keywords:** osteosutures, volar plate injury, fracture-dislocation, proximal interphalangeal joint, finger injury

## Abstract

The proximal interphalangeal (PIP) joint is the articulating joint between the proximal and middle phalanges of the fingers. A dorsal fracture-dislocation of the PIP joint of the fingers with volar plate injuries is an uncommon injury. Few cases have been published in the literature. In this article, we report the case of a subluxation fracture of the PIP joint in a 27-year-old male patient, without pathological history, a manual worker, right-handed, diagnosed 28 days after the injury. The treatment was surgical with open reduction and fixation of the fragment of the base of P2 with osteosutures. The functional results after three months were satisfactory with good sagittal and frontal joint stability and active flexion of the PIP joint at 95° and active extension at 0°. The control radiographs confirm the consolidation of the osteochondral fragment of the base of P2. The patient returned to his usual activities without pain.

## Introduction

The collateral ligaments ensure the frontal stability of the proximal interphalangeal (PIP) joint of the long fingers, which is a ginglymic joint. The palmar plate and the medial band of the extensor apparatus provide sagittal stability of the joint. The volar plate is frequently injured during PIP joint avulsion fractures [1]. The volar lip of the middle phalanx is where this type of avulsion fracture typically develops [2]. Dorsal fracture-luxations are lesions that if not diagnosed or incorrectly treated, can produce sequelae and severe functional impotence of the affected digital, causing stiffness, persistent subluxation, degenerative osteoarthritis, and chronic pain, hence the importance of early diagnosis and treatment of this type of injury. We present a case of a 27-year-old male patient, manual worker, right-handed who suffered a dorsal fracture-luxation of the PIP joint in the fifth finger of the right hand.

## Case presentation

**Figure 1:**
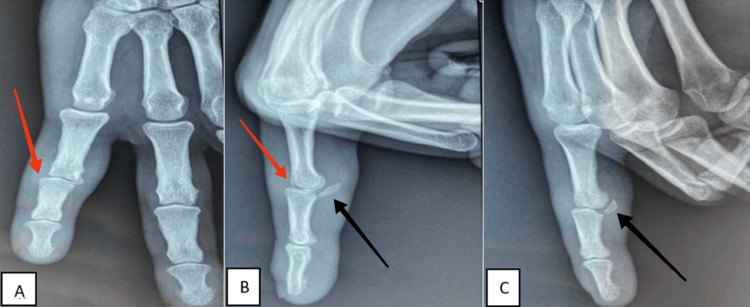
Standard radiographs anteroposterior (A), lateral (B) and lateral 3/4 (C) showing a dorsal subluxation of the PPI of the fifth right finger (red arrows) with bone avulsion of the base of the middle phalanx (black arrows).

A 27-year-old right-handed patient, a manual worker, without pathological history, and a victim of a traffic accident, was admitted to the emergency for pain and complete functional impotence with a wound of the PIP joint of the fifth finger of the right hand. The patient received a suture of the wound without clinical or radiological exploration. Three weeks after the trauma, the patient consulted our clinic due to the persistence of a deficit in active and passive flexion of the PIP of the fifth finger. The standard radiograph was in favor of a unicondylar fracture of the palmar base of the P2 classified as type four according to the classification of Weiss and Hastings, with a dorsal subluxation of the PIP (Figures 1A-1C).

The Weiss and Hastings classification system is used to categorize injuries of the volar plate at the PIP joint of the fingers. It was introduced by Weiss and Hastings in 1989. This classification system is based on the mechanism of injury and the severity of the volar plate disruption. Here is a brief explanation of the classification types: Type I (Incomplete Avulsion), Type II (Complete Avulsion), Type III (Distal Avulsion with Fracture), and Type IV (Fracture with Avulsion and Fragmentation).

**Figure 2:**
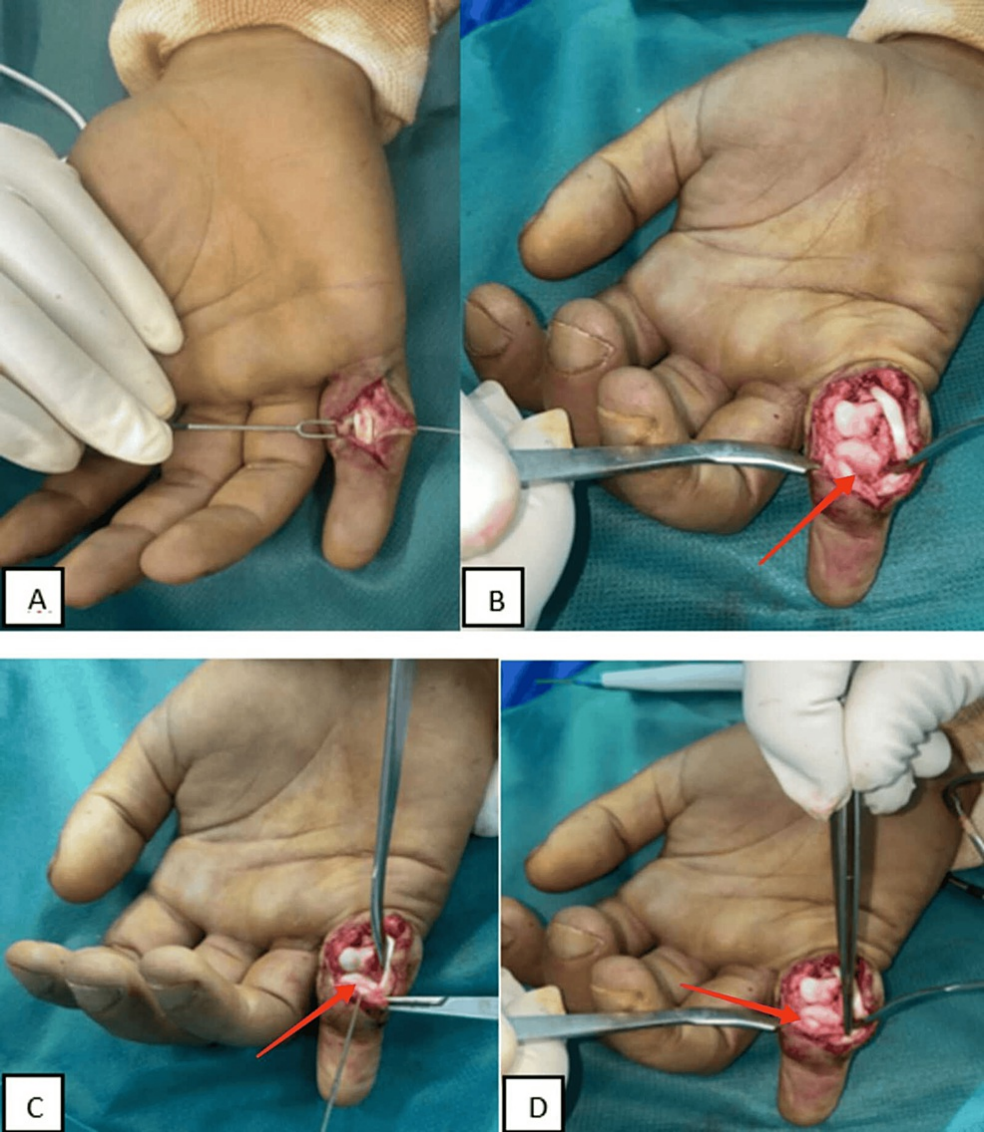
Intra-operative photograph showing (A) Bruner incision for the volar approach, (B) bone tear of the base of the middle phalanx, (C) temporary fixation of the bone fragment with a Kirschner wire, and (D) fixation of the bone fragment by osteosutures.

The diagnosis of fracture of the base of P2 and dorsal dislocation of the PIP of the fifth finger of the right hand was retained. The patient underwent open reduction and fixation; the incision was palmar (Bruner zigzag) on the PIP joint, after exploration of the digital canal, incision of the A3 pulley with lateral dislocation of the flexor apparatus, and disinsertion of the two lateral ligaments of the proximal phalanx, dislocation of the PIP joint (palmar shotgun approach). An osteochondral tear of the palmar plate opposite the base of P2 was discovered (Figures 2A-2D).

**Figure 3:**
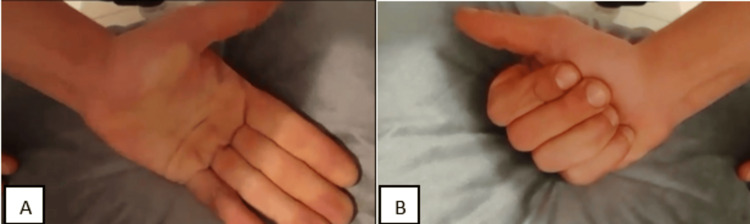
Clinical status after three months follow-up. (A) Active extension at 0°, (B) active flexion at 95°.

Osteosynthesis was performed after the reduction of the osteochondral fragment and transosseous reinsertion of the palmar plate using osteosutures. After satisfactory scopic control and stability testing, suture of the A3 pulley and then plane-by-plane closure. An adapted physical therapy protocol was established. At three months follow-up, the patient was pain-free, the range of active movement of the PIP was 95° in flexion and 0° in extension, and the sagittal and frontal stability of the PIP in flexion and extension was maintained (Figures 3A, 3B).

**Figure 4:**
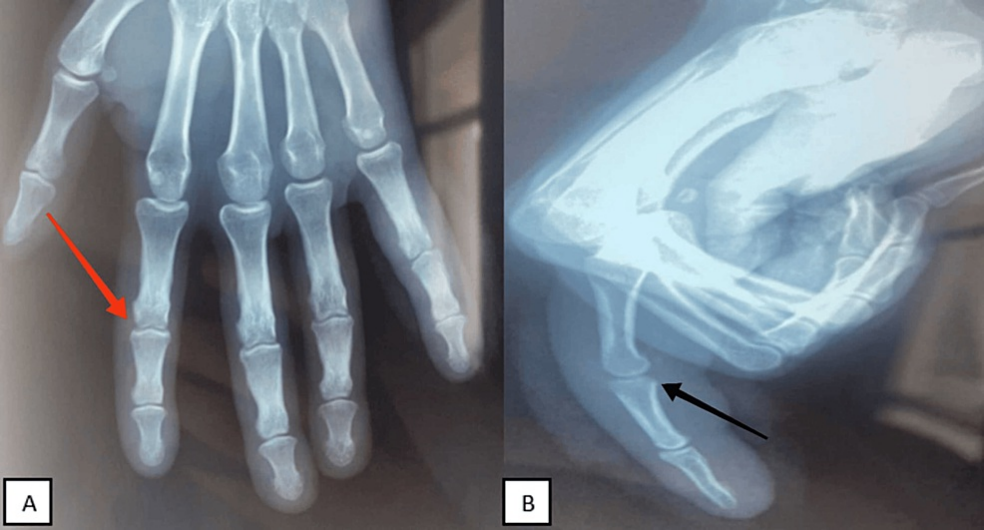
Radiological control ((A) anteroposterior, (B) lateral) showing good alignment of the fifth finger (red arrow) and consolidation of the osteochondral fracture site (black arrow).

The follow-up radiographs confirmed consolidation of the osteochondral fracture site (Figures 4A, 4B). The patient returned to his usual activities.

## Discussion

The fractures at the PIP joint are more frequently observed because of the leverage exerted by the last two phalanges, which amplify lateral or rotational pressures on the joint's surfaces [3]. The volar plate fails with forceful hyperextension of the finger by sprain, total rupture, or by avulsion of the middle phalangeal volar lip [4]. Because the check-rein ligaments have a stronger proximal attachment to the proximal phalanx, the volar plate typically detaches distally, as a result, injuries to the volar plate frequently occur during PIP joint avulsion [5]. A fracture might be as small as a bone flake or as large displaced fracture that affects more than half of the articular surface. Stable volar plate injuries refer to avulsion fractures that impact less than one-third of the articulating surface. The PIP joint becomes more unstable, and the risk of a volar plate injury increases with the size of the fracture fragment [6]. However, some small avulsion fractures may have developed as a result of a reduced prehospital finger dislocation. It is possible that such injuries x-rays do not always show the degree of initial deformity that existed prior to reduction [7].

A PIP joint subluxation could also involve a volar plate injury (VPI). VPIs are considered unstable when there are accompanying subluxations [1]. It has been demonstrated that identifying the “V-sign” on a true lateral radiograph can help diagnose these minor subluxations and consequently VPI. A V-sign is a radiographic anomaly that denotes an incongruent PIP joint and is characterized by an apparent dorsal crescentic gap [8]. The primary goal of treating PIP joint injuries is to establish a stable joint, which is essential for early mobilization and crucial in minimizing stiffness. Anatomic repair of the joint surface is a desired objective, but it is not the top concern; joint stability is attained by the lowering of the joint's volume, which permits sliding motion without hinging, which happens in joint subluxation and repair of the stabilizers, which could involve the PIP joint bony and soft tissue stabilizers [9].

Many different treatment approaches have been suggested for dorsal fracture dislocation of the PIP joint, such as extension locking techniques; this method involves the use of a double Zimmer splint contoured to the dorsum of the finger, leaving the wrist free, and specific flexion angles at the metacarpophalangeal and PIP joints. The splint is secured with adhesive tape, and the patient is encouraged to engage in active and passive finger flexion while extension is restricted by the splint [10,11], transarticular fixation; this method involves achieving congruent reduction of the joint, a 1.0 mm Kirschner wire is inserted into the bare area of bone on the dorsum of the middle phalanx and passed retrograde through the joint into the head of the proximal phalanx. An x-ray should be taken to confirm the concentric reduction of the PIP joint and immobilization with a Zimmer splint [12], static and dynamic external fixation. For this technique, the fracture was to be reduced by manual traction and manipulation, a smooth 1.2 mm Kirschner wire was then drilled through the center of the head of the proximal phalanx, perpendicular to its long axis, a second wire was then inserted parallel to the first through the head of the middle phalanx. The proximal wire was shaped into a tight hook on both sides of the finger. The distal wire was subsequently bent at a 90° angle in the frontal plane and contoured into a parabolic curve in the sagittal plane, directed towards the proximal wire. The wire ends are subsequently turned into secure hooks, which are interlocked with the hooks on the proximal wire after initially loading the wire curve. This process generates tension and distraction across the fracture [13,14], percutaneous or open reduction and internal fixation [15,16], palmar plate arthroplasty, and osteochondral bone grafting [17,18].

In our case, the presence of a rupture of the volar plate with avulsion of a bone fragment, a dorsal subluxation, and a deficit of active and passive flexion of the PIP joint an open reduction and fixation of the osteochondral fragment by osteosutures and reinsertion of the palmar plate has been realized. The patient underwent a gentle rehabilitation program starting in the first week to prevent joint stiffness, all while maintaining the immobilization splint for a duration of four weeks. It is advised to begin early mobilization for PIP joint fractures during the first week, and there is consensus in the literature that flexion activities for the PIP joint should be started shortly after injury, with extension exercises to follow two to three weeks later [19]. If the patient can flex their PIP joint at least 40 degrees in the first four weeks after an accident, the risk of tendon adhesions is greatly reduced [19].

The particularity of our case was the fact that it associated both a bone tear of the palmar plate with a displacement of the osteochondral fragment opposite the head of the proximal phalanx with subluxation of the PIP. By following the recommendations in the literature, we can effectively address the unique aspects of our case. First and foremost, employing a palmar approach is crucial as it allows for a comprehensive exposure of the palmar plate and the associated osteochondral tear. Additionally, fixation of the osteochondral fragment with respect to the articular surface. Lastly, initiating early rehabilitation is emphasized to promptly restore the appropriate mobility of the joint [20].

## Conclusions

Fracture-dislocation dorsal of the PIP joint is an uncommon injury. Maintaining motion and function after injury of the PIP joint is still highly difficult due to its restricted bone congruity and surrounding soft tissues. This surgical technique of open reduction of dorsal fracture-dislocation of the PIP joint of the fingers with volar plate injuries can successfully achieve a functional range of motion with a stable joint.
